# Structural Integrity of the Antigen Is a Determinant for the Induction of T-Helper Type-1 Immunity in Mice by Gene Gun Vaccines against E.coli Beta-Galactosidase

**DOI:** 10.1371/journal.pone.0102280

**Published:** 2014-07-15

**Authors:** Tekalign Deressa, Angelika Stoecklinger, Michael Wallner, Martin Himly, Stefan Kofler, Katrina Hainz, Hans Brandstetter, Josef Thalhamer, Peter Hammerl

**Affiliations:** Department of Molecular Biology, University of Salzburg, Salzburg, Austria; University of Massachusetts Medical Center, United States of America

## Abstract

The type of immune response is critical for successful protection and typically determined by pathogen-associated danger molecules. In contrast, protein antigens are usually regarded as passive target structures. Here, we provide evidence that the structure of the antigen can profoundly influence the type of response that is elicited under else identical conditions. In mice, gene gun vaccines induce predominantly Th2-biased immune reactions against most antigens. One exception is E. coli beta-galactosidase (βGal) that induces a balanced Th1/Th2 response. Because both, the delivered material (plasmid DNA-coated gold particles) as well as the procedure (biolistic delivery to the skin surface) is the same as for other antigens we hypothesized that Th1 induction could be a function of βGal protein expressed in transfected cells. To test this we examined gene gun vaccines encoding structural or functional variants of the antigen. Employing a series of gene gun vaccines encoding individual structural domains of βGal, we found that neither of them induced IgG2a antibodies. Even disruption of the homo-tetramer association of the native protein by deletion of a few N-terminal amino acids was sufficient to abrogate IgG2a production. However, enzymatically inactive βGal with only one point mutation in the catalytic center retained the ability to induce Th1 reactions. Thus, structural but not functional integrity of the antigen must be retained for Th1 induction. βGal is not a Th1 adjuvant in the classical sense because neither were βGal-transgenic ROSA26 mice particularly Th1-biased nor did co-administration of a βGal-encoding plasmid induce IgG2a against other antigens. Despite this, gene gun vaccines elicited Th1 reactions to antigens fused to the open reading frame of βGal. We interpret these findings as evidence that different skin-borne antigens may be differentially handled by the immune system and that the three-dimensional structure of an antigen is an important determinant for this.

## Introduction

For the establishment of protective immunity the type of effector mechanism is a decisive determinant. E.g., cytotoxic T lymphocytes (CTL) may be effective against intracellular infections, Th1 (but not Th2) reactions against leishmaniasis or lepra [1], [2], [3], [4], and different types of immunoglobulins are differentially involved in a diverse set of defense mechanisms such as phagocytosis, mast cell degranulation or complement activation [5], [6]. The decision for a particular IgG subclass is determined by the type of T cell help which, in turn, is shaped by the interactions of naïve T cells with antigen-presenting cells (APC), particularly dendritic cells (DC). Evolution of vertebrates in a microbial environment has equipped APC with a large panel of receptors that recognize a broad range of highly diverse microbial substances, commonly referred to as pathogen-associated molecular patterns (PAMPS) [7], [8]. Differential DC activation by PAMPS profoundly influences the type of T cell polarization, inflammatory reactions and other downstream events [9], [10]. While PAMPS, endogenous immune modulators and host-pathogen interactions have been intensively studied, immune modulating activities of protein antigens are much less appreciated. However, current attempts to develop saver vaccines by substituting purified or recombinant antigens for attenuated pathogens urge a better understanding of direct effects of antigens on the shaping of immune reactions. So far, only few protein antigens with immune modulatory activity have been described. E.g., certain proteases from house dust mites or papain induced allergic Th2 reactions in the absence of any adjuvant [11], [12]. The house dust mite allergen Der p 2 induced allergic asthma by mimicking the effect of the LPS-binding Tlr-4 co-receptor MD-2 [13]. It has also been recognized that biochemical parameters such as the stability of protein folds and accessibility to lysosomal proteolysis can influence immunogenicity [14], [15]. All these examples are extracellular antigens. However, in view of the serious threats imposed by intracellular pathogens such as viruses, but also tumor antigens, it might be of relevance to understand the influence of host cell-derived antigens on immune modulation.

The investigation of direct immune modulation by protein antigens requires the exclusion of modulatory influences that are superimposed by strong adjuvants. However, injection of soluble proteins without adjuvants does usually not yield an efficient immune response, and the problem of residual bacterial contaminations in recombinant proteins remains an issue. In contrast, DNA vaccines induce robust cellular and humoral immune reactions even when administered without the addition of immune stimulating adjuvants. Typical DNA vaccines are expression plasmids that encode the antigen of interest driven by a strong eukaryotic or viral promoter. Upon immunization, host cells are transfected in vivo and start to produce the antigen, much like in the initial processes in viral infections [16]. The mechanisms that lead to the activation of the immune system by this procedure are not well known. A popular hypothesis is based on the immune stimulating features of bacterial DNA [17], [18], but DNA vaccines retained their immunogenicity in Tlr9-deficient mice [19]. Alternative explanations involve the activation of TBK-1 with downstream induction of type-1 interferon signaling pathways [20].

In mice, epicutaneous bombardment with DNA-coated gold particles by means of a gene gun device elicits predominantly Th2 reactions, indicated by the induction of IgG1 but not IgG2a antibodies [21], [22], [23]. One exception is gene gun vaccines encoding beta-galactosidase (βGal) from E. coli that induce both antibody isotypes [24], [25], [26]. For gene gun vaccines such differences between antigens are remarkable because, firstly, the substance delivered to the host (i.e. plasmid DNA) is the same as for other antigens and, second, the conditions of vaccine delivery are also identical. Thus, the only obvious differences are at the level of the antigen protein produced by the host cells. Therefore, we hypothesized that the Th 1-favoring immune modulating activities of βGal gene gun vaccines could depend on the structure or function of the antigen gene product.

βGal is a non-covalently associated homo-tetramer of 120 kDa polypeptide subunits that comprise five well-defined structural domains. The tetramer is assembled by head-to-head contacts between domain-1 surface elements of adjacent subunits and tail-to-tail contacts made up by domain-5 residues [27]. The tetrameric organization is essential for the catalytic activity of this beta-D-galactoside hydrolyzing enzyme. Deletion of a few N-terminal amino acid residues is sufficient to dissociate the tetrameric structure and abrogate the enzymatic activity [28]. The present study aimed at investigating which structural elements or features of βGal could be responsible for the Th1-type immune response elicited by gene gun vaccination.

## Materials and Methods

### Mice

BALB/cAnN and C57Bl/6N mice were purchased from Charles River, Sulzfeld, Germany. IL4^−/−^ [29], IFNγ^−/−^ [30] and IL12p40^−/−^ [31] and ROSA26 [32] mice were obtained from the Jackson Laboratory, Bar Harbor, Maine, USA. Tlr4-deficient C57Bl/10ScCR mice were a kind gift of Drs. M. Freudenberg and C. Galanos, Freiburg, Germany [33]. Colonies of these strains were maintained at the local animal facility under specific pathogen-free conditions [34]. Stable groups of 4 to 6 females per cage were used between 6 to 10 weeks (wild types) or 6 to 16 weeks of age (transgenics) at the start of experiments. Animals were kept in individually ventilated cages (Type II long, Tecniplast, Germany) at 65 air changes per hour maintaining positive cage pressure, with access to sterilized chow and autoclaved water *ad libitum*. Fresh cages with woodchip bedding and paper-based nesting material, all autoclaved, were provided weekly. Animal rooms were kept at 20–22°C and 45–65% relative humidity with automated light/dark periods of 12/12 hrs. Animals are daily inspected for any signs of discomfort.

### Ethics Statement

Animal experiments were conducted in accordance with EU guidelines 86/609/EWG and national legal regulations (TVG 2012) and all efforts were made to minimize or avoid suffering. Experiments were approved by the Austrian Ministry of Science, Ref. II/3b (Gentechnik und Tierversuche), permission no. GZ66.012/0014-II/10b/2010.

### Vaccine plasmids

Gene gun vaccine plasmid constructs were all based on the eukaryotic expression vector pCI (Promega, Madison, WI). pCI-βGal and pCI-OVA have been described [26], [35]. The enzymatically inactive mutant βGal E537A was generated by PCR-based site-directed mutagenesis substituting alanine for the catalytic center glutamate in position 537. Coding sequences for βGal domains (D1: M1-P219, D2: T220-R334, D3: E335-Q626, D4: F627-T730 and D5: L731-K1024; numbering according to NCBI Acc. No. YP_008570279) were amplified by PCR with sense primers providing a Kozak consensus sequence including a start codon (GCCACCATG) and cloned into the unique EcoRI and SalI sites of pCI. Delta-βGal, a construct with deletion of the N-terminus (M1-E41) was generated by PCR-based site-directed mutagenesis with a sense primer providing a Kozak sequence as above. pCI-based plasmid constructs for fusion proteins cOVA-βGal, OVA-βGal, βGal-OVA, EGFP-OVA and mCherry-OVA [36] were generated by PCR techniques, abutting the open reading frame (ORF) for the C-terminal protein right behind the last codon of the N-terminal fusion partner of which the stop codon had been deleted. cOVA, a deletion mutant of OVA lacking the secretory signal peptide, was generated by deletion of AA 20–145 employing SacI cleavage and religation [37]. Plasmids for the house dust mite allergen Der p 2 (GenBank Acc. No. DQ185510.1), the cat allergen Fel d 1, constructed as a fusion of both polypeptide chains [38] (GenBank Acc. No. chain 1, NM_001048153; chain 2, M77341.1), and the timothy grass allergen Phl p 6 (GenBank Acc. No. Y16956) were kindly provided by Dr. R. Weiss from our department. The open reading frames were excised with restriction enzymes EcoRI and NotI and cloned into the unique EcoRI and NotI sites of pCI.

### Recombinant proteins

ORFs for βGal domains D1–D5 were excised from the vaccine vectors by EcoRI/SalI digestion and ligated into the unique EcoRI/SalI sites of plasmid pMPB parallel II (New England Biolabs Inc.) to generate fusion proteins with an N-terminal maltose binding protein (MBP) tag for purification. 6×His-tagged βGal was expressed and purified by Ni-chelate affinity chromatography from pET-28 βGal control vector (Novagen). The ORF for delta-βGal was excised from pCI-delta-βGal by NcoI/NotI digestion and ligated into NcoI/NotI sites of pHIS-parallel II (Novagen). Proteins were expressed in E. coli BL21(DE3) and purified by nickel- or maltose affinity chromatography, respectively.

### Immunization and blood sampling

Gene Gun immunization was performed as described [39]. One dose comprised two non-overlapping shots onto the shaved abdominal skin. With each shot, 1 µg of plasmid DNA, immobilized onto 0.5 mg gold particles, was delivered with pressurized helium gas at 400 psi using a Helios gene gun (Bio-Rad, Richmond, CA). Potential stress caused by noise from the gene gun shots was minimized by immunizing animals in a remote laboratory. Immunization with proteins was carried out by intradermal injection of 10 µg protein in 100 µL pyrogen-free PBS, distributed to 4 portions applied to the ventral abdominal skin. Blood samples were collected by tail vein puncture after pre-warming the tail in a 42–45°C water bath.

### ELISA

Antigen-specific serum antibodies were detected by ELISA with MBP- or 6×His-tagged recombinant antigens immobilized on NUNC-Maxisorp ELISA plates, using isotype-specific peroxidase-conjugated detection Abs, followed by chromogenic development. Antibody titers were determined by endpoint titration and expressed as the dilution factor yielding a response equal to the quantification limit (i.e., mean+3×SD of 8–16 blank values). IFNγ in supernatants of in-vitro restimulated spleen cells were measured by sandwich ELISA with capture antibody (clone AN-18) and biotinylated detection antibody (clone R4-6A2) followed by streptavidin-conjugated peroxidase (all from BioLegend) and chromogenic development.

### In vivo-CTL assays

In vivo-CTL assays were carried out as described [40]. Briefly, a mixture of CTL peptide-pulsed and non-pulsed syngenic target cells, stained with differential concentrations of CFSE, was injected i.v. into immunized mice. Next day, spleens of recipients were analyzed by flow cytometry and specific lysis was calculated from the ratio of peptide-pulsed target cells to non-pulsed reference cells.

### ELISPOT assays

ELISPOT assays were carried out as described [40]. Briefly, 2×10^5^ spleen cells/well were re-stimulated in filter-bottom plates, pre-coated with cytokine-specific capture antibody, with 10 µg/mL CTL peptide (DAPIYTNV for βGal, SIINFEKL for OVA) or 20 µg/mL of protein for 20 hrs. Cytokine spots were detected with biotinylated cytokine-specific detection antibody and peroxidase-conjugated streptavidin, followed by chromogenic enzyme reaction.

### Cell transfection and enzyme assays

BHK21 cells (ATCC: CLL-10) were grown in DMEM supplemented with 5% heat-inactivated FCS, 1 mM Na-pyruvate, 4 mM L-Gln, 10 mM HEPES and antibiotics. Cells were transfected with the indicated plasmids with Metafectene (Biontex, Germany) according to the manufacturer's instructions, cultured for an additional 24 hours. For luciferase or galactosidase enzyme activity cell lysates were analyzed by bioluminescence assays with Galacto-Star (Life Technologies) and Luciferase Reporter Assay kits (Promega), respectively. Signals were recorded on an Infinite-200 multireader (Tecan, Austria).

### Size exclusion chromatography

The molecular weight of βGal and delta-βGal expressed from vaccine plasmids was determined by size exclusion chromatography of lysates prepared in PBS from BHK-21 cells transfected with pCI-βGal or pCI-delta-βGal, respectively. Lysates were separated on a Superose-6 column (GE Healthcare Life Sciences). Eluted fractions were used to coat ELISA plates and βGal-related protein was detected by ELISA with a murine anti-βGal antiserum and peroxidase-conjugated anti-mouse IgG detection antibody. Recombinant delta-βGal expressed and purified from E. coli was analyzed by high performance size exclusion chromatography on a TSKgel G2000(SWXL) 7.8×300 mm column (Tosoh Bioscience) in 100 mM Na-phosphate buffer, pH 6.6.

### Circular Dichroism (CD) spectroscopy

CD spectra of recombinant βGal and delta-βGal expressed and purified from E. coli were recorded from 0.1 mM protein solutions in 10 mM potassium phosphate buffer, pH 7.4, on a J-810S spectropolarimeter (Jasco, Germany). The mean residue ellipticity ([θ]m.r.w.) was calculated from the measured ellipticity [θ] as described [41].

### Dynamic Light scattering (DLS) analysis

DLS spectra of recombinant βGal and delta-βGal expressed and purified from E. coli were recorded from 1 mg/mL protein solutions in 10 mM potassium phosphate buffer, pH 7.4, on a DLS 802 instrument (Viscotek Corp., TX, USA). Samples were centrifuged at 14.000× g from 10 min prior to analysis of the supernatant. Data were accumulated for 10×10 sec and the correlation function was fitted into the combined data curve, from which the intensity distribution was calculated and transformed to mass distribution.

### Microsomal protease degradation of antigens

Recombinant βGal and delta-βGal expressed and purified from E. coli were analyzed for susceptibility to proteolysis. To this end, microsomal fractions from JAW II dendritic cell-like cells (ATCC: CRL 11904) were isolated by disruption of cells in 10 mM Tris/acetate buffer, pH 7, with 250 mM sucrose with a Dounce tissue homogenizer. Nuclei were removed by centrifugation (6.000× g for 10 min) and microsomes were harvested by ultracentrifugation at 100.000× g for 1 hour. Pellets were lysed in the above buffer by 5 freeze/thaw cycles in liquid nitrogen, centrifuged and stored frozen until use. Five µg of recombinant antigens were added to 20 µg microsomal enzyme preparation in 100 mM citrate buffer, pH 4.8, 2 mM DTT, and incubated at 37°C for the indicated periods of time. Reactions were stopped by addition of SDS sample buffer and heating to 95°C for 5 min. Antigen degradation was analyzed by SDS-PAGE and densitometry of Coomassie Blue-stained gels.

### Statistical analysis

ELISA, ELISPOT and CTL Data were plotted as means with standard deviations. Statistical significance was calculated by two-tailed Student's t-test assuming unequal variance. Comparisons with p-values<0.05 were considered statistically significant.

## Results

### Gene gun vaccines encoding βGal induced IgG2a in a Th1-dependent manner

**Figure 1:**
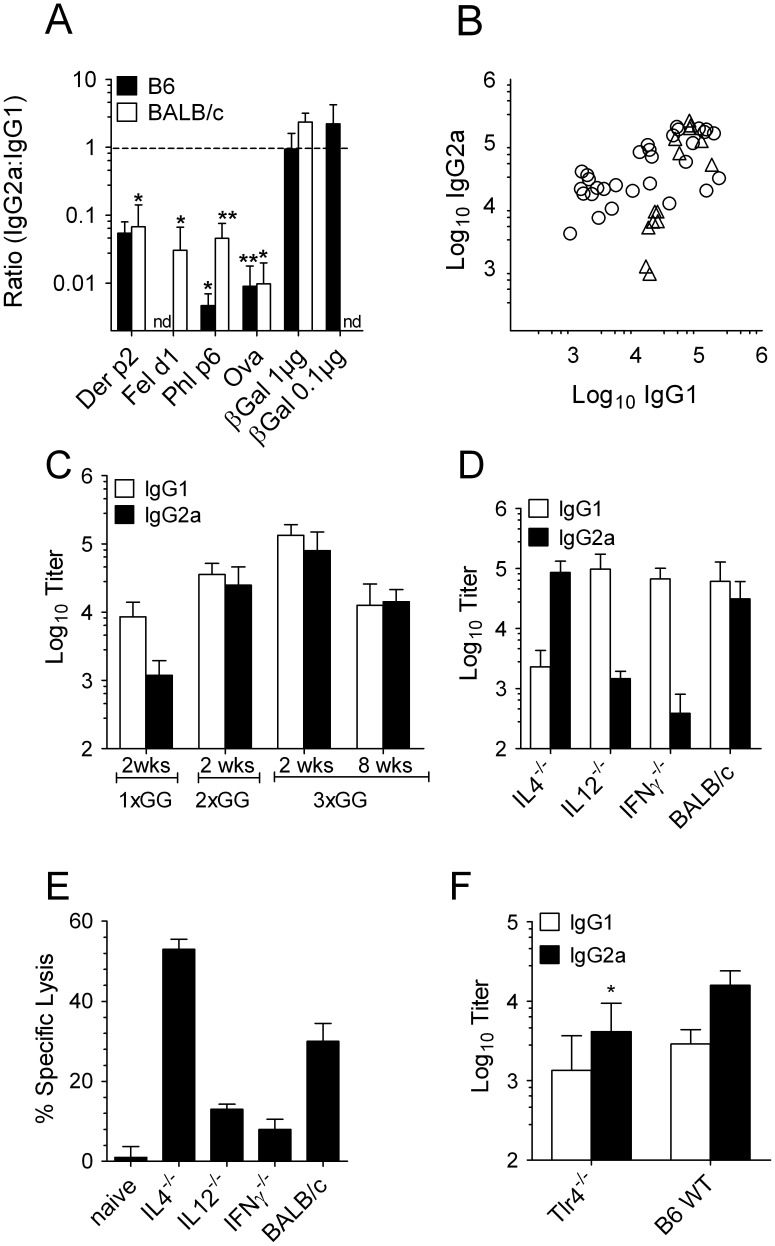
Unlike other antigens, βGal gene gun vaccines induced a balanced Th1/2 antibody response. (A) Ratio of antigen-specific IgG2a:IgG1 in B6 and BALB/c mice (n = 3 to 8) two weeks after two gene gun immunizations with plasmid vaccines encoding different antigens administered at 2-week intervals. With each shot, 1 µg of plasmid was administered for all antigens; pCI-βGal was additionally tested with a dose of 100 ng/shot. *(p<0,05) and **(p<0,01) indicate groups that elicited significantly less IgG1 than IgG2a. (B) anti-βGal IgG1 and IgG2a in individual B6 (circles) or BALB/c (triangles) mice after gene gun immunization with pCI-βGal. Cumulative data from 8 independent experiments. (C) anti-βGal serum IgG1 and IgG2a in B6 mice (n = 5) 2 weeks after one (1×GG) or two gene gun immunizations (2×GG), and 2 or 8 weeks after a third immunization (3×GG). (D) IgG isotype titers and (E) cytotoxic activity against βGal in cytokine knockouts on BALB/c background (groups of n = 5, each). (F) anti-βGal serum IgG1 and IgG2a 2 weeks after 2 gene gun shots in Tlr4-deficient B10ScCR and in B6 wt mice. *, p<0,05 vs. WT.

In previous projects, we found that βGal is an unusual antigen in that it induced multiple IgG isotypes including IgG1, 2a/c and IgG2b. This was even true for BALB/c mice that are genetically biased towards Th2-polarized immunity [42]. To demonstrate this, we immunized B6 and BALB/c mice with gene gun vaccines encoding different antigens. These included the house dust mite allergen Der p 2, a 129 amino acid (AA) beta-barrel protein with homology to the Tlr4 co-receptor MD-2 [13], [43], the cat saliva allergen Fel d 1, a tetramer composed of 2 identical heterodimers of 92 (chain A) and 109 AA (chain B) α-helical polypeptides [44], Phl p 6, a 110 AA α-helical Zn-binding polypeptide (protein data bank PDB: 1NLX) [45] and hen egg albumin (OVA), a member of the serpin superfamily of 386 AA [46]. pCI-βGal induced comparable amounts of IgG1 and IgG2a whereas with other antigens IgG2a∶IgG1 ratios were only low. The induction of IgG2a against βGal did not simply originate from a high vaccine dose as (i) all vaccines tested contained the same amount of plasmid and (ii) IgG2a was also induced with a pCI-βGal vaccine containing a 10-fold lower dose of the plasmid (fig. 1A). Production of both isotypes was observed in each individual through a series of independent experiments performed in, either, B6 or BALB/c mice (fig. 1B). IgG2a was not only detected in early antisera but persisted over eight weeks after the last of three gene gun immunizations (fig. 1C). As expected, the production of IgG2a was classically dependent on Th1 cytokines, as mouse strains deficient in, either, IL12p40 or IFNγ induced this isotype only to 1% or less as compared to wild type mice. Conversely, IL4 knockout mice were almost unable to produce IgG1 whereas IgG2a production was unaffected (fig. 1D). The influence of the Th1/2 cytokine balance was also reflected at the level of CD8^+^ T cells. Compared to WT mice CTL were lower, albeit not completely abrogated, in IL12p40 and IFNγ knockout mice and higher in IL4-deficient mice (fig. 1E). The induction of Th1 cytokines, particularly IL12, could be a consequence of toll-like receptor (Tlr) signaling induced by microbial matter. E.g. LPS, which is biologically active at extremely low concentrations, might be introduced from the skin surface with the penetration of the epidermis by gene gun gold particles. However, Tlr4-deficient mice still induced a pattern of IgG1/2a ratio that was comparable to that of WT mice (fig. 1F).

### Isolated structural domains of βGal did not induce IgG2a

**Figure 2:**
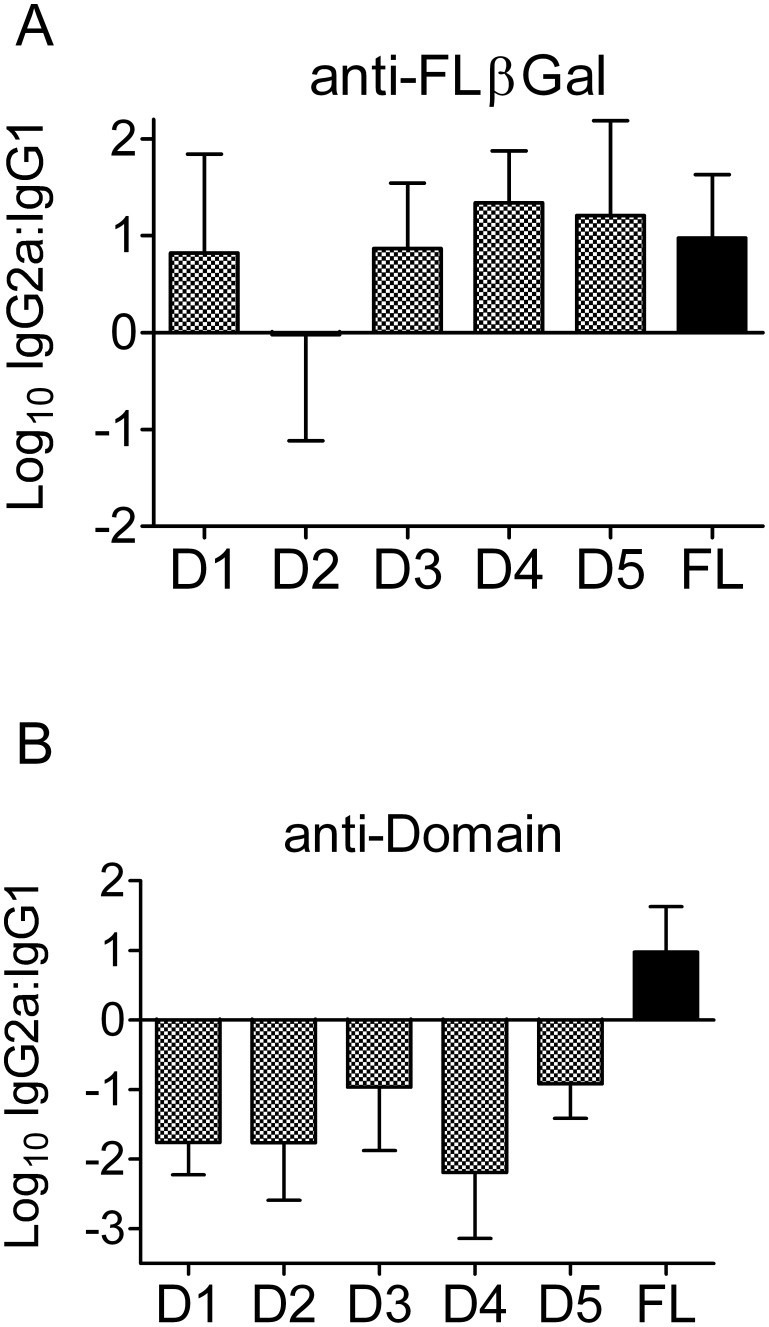
Gene gun vaccines encoding individual domains elicited predominately Th2-associated IgG1 antibodies. (A) Sera from B6 mice (n = 5) gene gun-immunized with pCI-βGal twice at a 14 d interval, tested by ELISA on recombinant βGal domains or, for comparison, full length βGal (FL). (B) Sera from B6 mice (n = 4–5) gene gun-immunized with plasmids encoding individual βGal domains D1–D5, or full length βGal (FL), respectively, tested on full length βGal-coated ELISA plate wells. Mice were immunized twice at a 14 d interval and sera were collected 14 d after the boost. Diagrams present means +/− s.d. of log(10) isotype ratios of IgG2a:IgG1, i.e. positive values indicate predominating IgG2a and, hence, Th1-biased reactions.

The induction of IgG2a antibodies in mouse is promoted by Th1 cells that, in turn, require appropriate activation by DC. Given the lack of adjuvant compounds in gene gun vaccines, we hypothesized that βGal itself could deliver a maturation signal to DC. We speculated whether such signaling motifs could reside in a particular structural domain of βGal. To address this question, we immunized B6 mice with gene gun vaccines encoding isolated structural domains of βGal and compared the antibody response to that obtained with the full length antigen. Antibodies raised against full length βGal targeted preferentially domains 1, 4, and 5 when tested by ELISA on recombinantly expressed individual domains. However, each of these domains bound more IgG2a than IgG1; no domain could be identified that was preferentially targeted by, either, IgG1 or IgG2a antibodies, respectively (fig. 2A). In contrast, when mice were immunized with gene gun vaccines encoding isolated structural domains of βGal, all domains elicited a clear Th2-type antibody spectrum with highly predominating IgG1 titers, whereas IgG2a titers were mostly close to or below the detection limit (fig. 2B). Thus, because isolated domains were *per se* unable to induce a Th1 response, it seemed unlikely that the Th1 bias of the full length antigen originated from a particular structural motif of the antigen.

### Disruption of the tetrameric organization of the immunogen abrogated IgG2a induction

**Figure 3:**
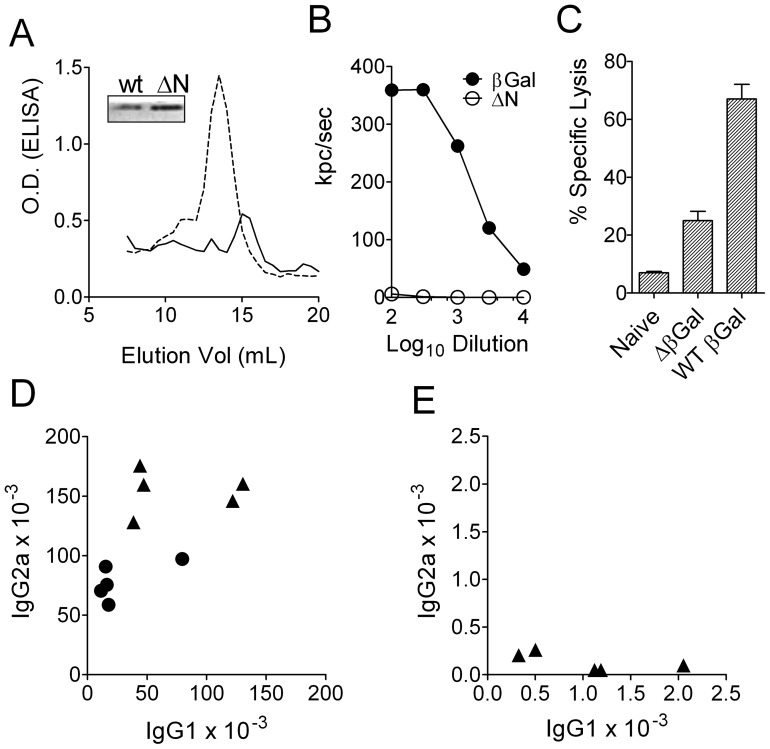
Disruption of the tetrameric structure of βGal reduced immunogenicity and abrogated IgG2a production. (A) Size exclusion chromatography and Western Blot (inset) of lysates of BHK21 cells (ATCC CCL-10) transfected with, either, wild type (wt, dashed line) or N-terminally truncated ΔβGal (ΔN, solid line); fractions analyzed for βGal by ELISA. (B) Enzymatic βGal activity of serially diluted lysates of transfected cells (from samples shown in fig. 3A inset) as determined by luminescence and expressed as kilo-photon counts (kpc) per second. (C) in-vivo CTL activity in B6 mice (n = 5) 14 d after 2 gene gun immunizations with pCI-ΔβGal or the full length wild type sequence (WT βGal). (D,E) Serum IgG isotypes of individual mice, 2 weeks after two gene gun immunizations with pCI-βGal (D) or pCI-ΔβGal (E).

**Figure 4:**
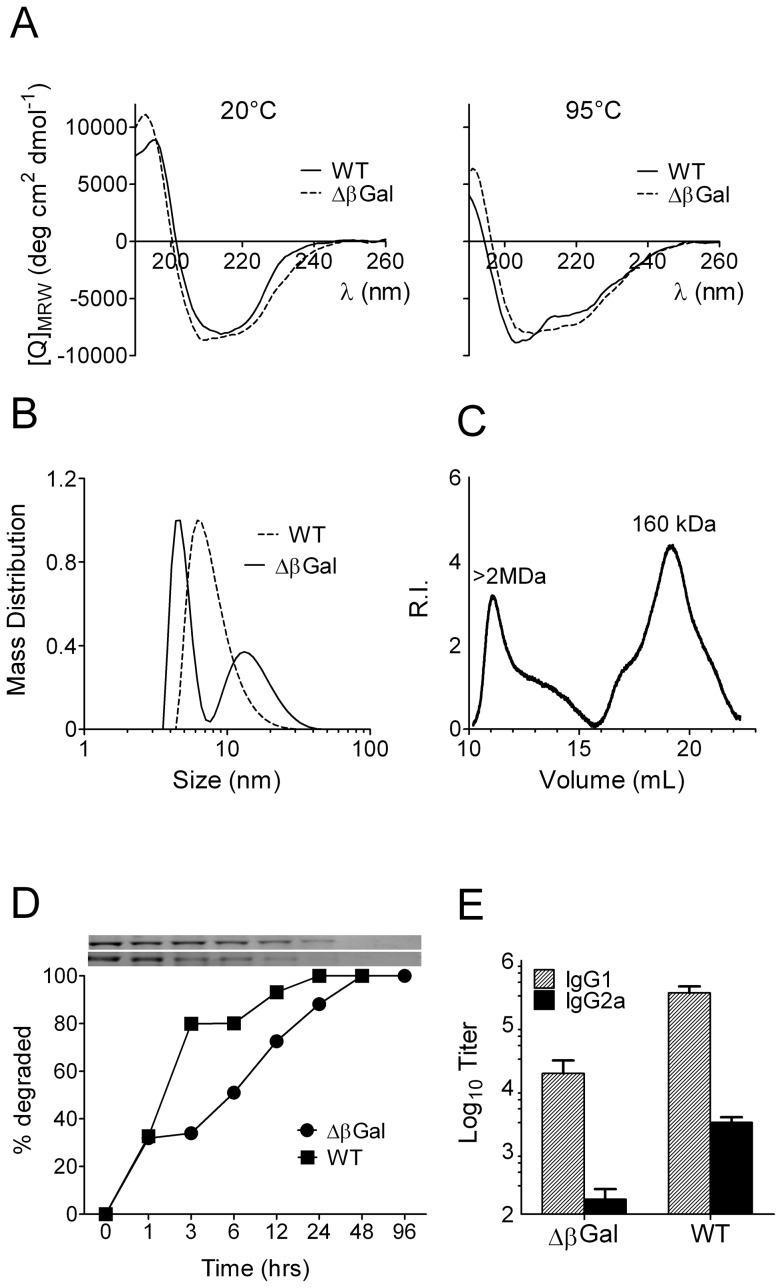
Comparison of recombinant 6×HIS-tagged full length wild type βGal and N-terminally truncated ΔβGal. (A) Circular dichroism spectra recorded at 20°C (left) or at 95°C (right). (B) Dynamic light scattering analysis of full length (WT) and truncated 6×HIS-tagged ΔβGal. (C) Size exclusion chromatography of the truncated ΔβGal protein. (D) Time course of microsomal degradation of full length (WT) βGal and truncated 6×HIS-tagged ΔβGal, calculated from densitometric analysis of SDS-PAGE samples drawn at the indicated time points (inset). (E) Serum IgG elicited by full length βGal (WT) or truncated 6×HIS-tagged ΔβGal. 10 µg were injected i.d. without adjuvant in groups of B6 mice (n = 5) twice at a 14 d interval. Sera were collected 2 weeks after the boost.

Because none of the isolated domains of the βGal monomer was able to induce IgG2a we hypothesized that a more complex structural entity of the antigen could be required for Th1 induction. Native βGal is a tetramer of 4 identical polypeptides of 1023 amino acids, each. To test whether the tetramer organization was required for the induction of a Th1 response, we generated a gene gun vaccine, pCI-delta-βGal, in which 40 residues were deleted from the N-terminus of the full length polypeptide. This region includes the so-called alpha peptide of βGal, and deletion of the alpha peptide disrupts the tetrameric association of the native protein [28]. For initial characterization of this construct BHK cells were transfected in-vitro. Size exclusion chromatography of crude lysates of BHK cells transfected with pCI-βGal revealed a major peak of anti-βGal-reactive protein at approximately 450 kDa, consistent with the tetrameric composition of the native protein. In contrast, cells transfected with pCI-delta-βGal expressed anti-βGal-reactive protein that eluted at approximately 220 kDa, suggesting a dimeric association of βGal polypeptides (fig. 3A). Deletion of the alpha peptide did not affect gene expression and/or stability of the truncated polypeptide, delta-βGal, in transfected BHK cells (fig. 3A inset). However, because the tetrameric organization is required for enzyme activity the truncated molecule was unable to hydrolyze galactoside substrates (fig. 3B). Despite unaffected protein synthesis in transfected cells, the immunogenicity of a gene gun vaccine encoding the truncated version, pCI-delta-βGal, was strongly decreased. This was observed with CTL activity (fig. 3C) and even more at the level of antibodies which were only about 2% of that elicited with the full length vaccine (fig. 3D, E). In contrast to the full length vaccine, delta-βGal elicited a clear Th2-related antibody isotype spectrum, i.e. only IgG1 but no IgG2a/c antibodies were detectable. In view of the great differences in both, immunogenicity and IgG isotype spectrum caused by the deletion of only a few amino acid residues, structural features of the truncated polypeptide were further investigated. Circular dichroism spectra of recombinant delta-βGal recorded at 20°C or 95°C were similar to those obtained with full-length βGal, indicating that secondary structures and their thermal stability were almost unaffected by the deletion of the alpha peptide (fig. 4A). Dynamic light scattering (DLS) analysis of the full-length protein showed a single population with a hydrodynamic radius (R_H_) of R_H_ = 6.3 nm, corresponding to the size of the native tetrameric protein. In contrast, delta-βGal revealed two major populations, one with a R_H_ value of 4.8 nm, consistent with a polypeptide dimer, and a second one at R_H_ = 15.5 nm (range 8–25 nm), indicating the presence of high molecular weight aggregates (fig. 4B). The presence of aggregates was also observed by size exclusion chromatography of recombinant delta-βGal (fig. 4C). The sensitivity of full-length and delta-βGal to antigen-processing proteases was examined by exposing both proteins in vitro to microsomal extracts isolated from dendritic cells. Recombinant delta-βGal was more resistant to proteolytic degradation than WT-βGal. Under the chosen conditions, the half-life time of full length βGal was slightly above 1 hour, whereas that of delta-βGal was approximately 6 hours (fig. 4D). To investigate the immunogenicity of both antigen variants, mice were immunized with 10 µg of the purified recombinant proteins. The addition of adjuvants was omitted to exclude T cell-polarizing activities that might interfere with those of the antigens themselves. Similarly to gene gun vaccines, recombinant delta-βGal was significantly less immunogenic than full-length βGal and elicited only 10% of the antibody titers that were induced with the latter. However, the isotype spectra were virtually identical and dominated by IgG1, whereas IgG2a was two orders of magnitude lower (fig. 4E). Together, the physic-chemical characterization of delta-βGal suggested that the overall structure of the polypeptide was at least similar to that in the native protein. However, the truncated molecule appeared to form high molecular weight aggregates and, perhaps as a consequence of this, was more resistant to lysosomal degradation. By limiting the production of antigenic peptides resistance to degradation might also account for the observed decrease in immunogenicity, as previously reported for hen egg lysozyme mutants with increased stability [15].

### Enzymatic activity of βGal is not required for the induction of IgG2a

**Figure 5:**
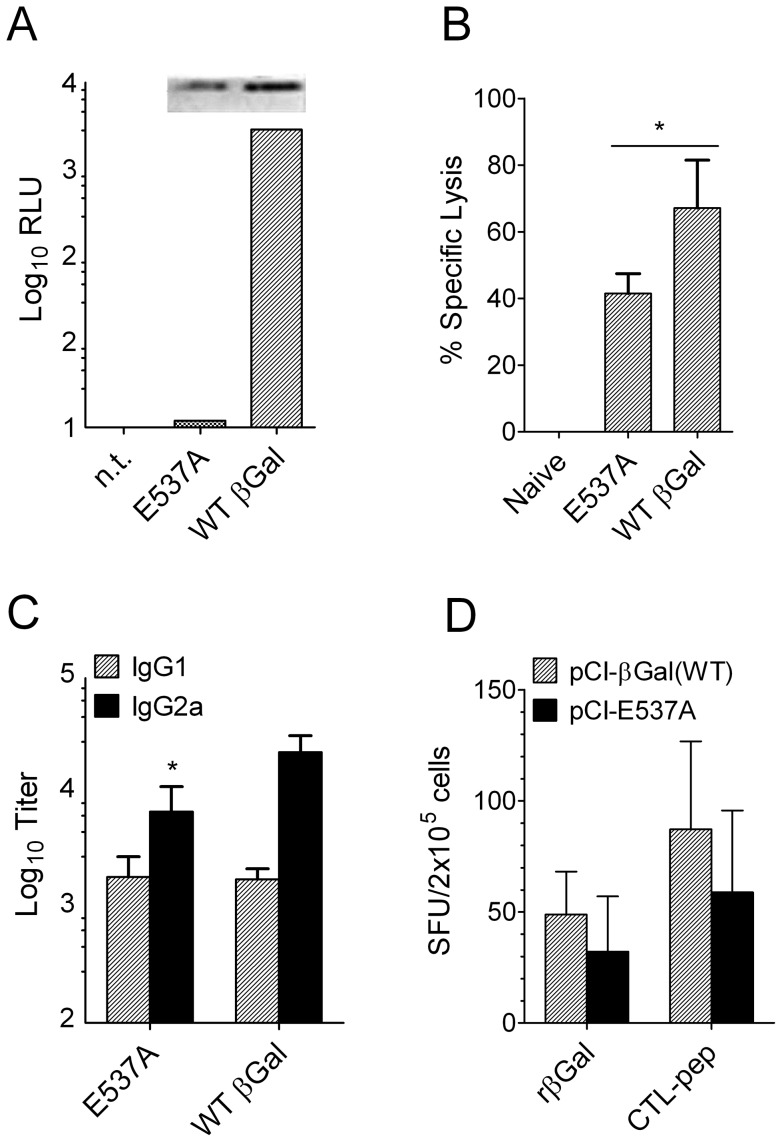
Loss of enzymatic activity of βGal did not influence the type of immune response. (A) Enzymatic activity of wild type (WT) βGal and the E537A mutant in transfected BHK21 cells, measured by luminogenic substrate hydrolysis and expressed as relative light units (RLU). Inset: Western blot of cell lysates (left: E537A, right: wild type βGal). (B) in vivo CTL assay and (C) Serum IgG in B6 mice (n = 5) 2 weeks after the second of 2 gene gun immunizations with, either, pCI-βGal (WT) or pCI-E537A-βGal. *, p<0.05 vs. WT. (D) Frequency of IFNγ-producing spleen cells of mice shown in (B, C) after restimulation in-vitro with either recombinant βGal protein or CTL-peptide.

Glycan recognition by lectins such as galectins is involved in many biological processes including immune cell activation and homeostasis [47]. βGal hydrolyzes β-D-glycosidic bonds in galactosyl compounds with broad substrate specificity. Therefore, we hypothesized that enzymatically active βGal could alter immune functions so that gene gun vaccines will induce also Th1 reactions. We engineered the βGal coding sequence by site-directed mutagenesis to substitute alanine for the nucleophilic residue glutamic acid 537 in the catalytic center. The mutant molecule (βGal E537A) was expressed in transfected BHK cells with an efficacy similar to the wild type molecule but did not show enzymatic activity (fig. 5A). Mice immunized with a gene gun vaccine for the inactive βGal mutant (pCI-E537A) induced βGal-specific CTL (fig. 5B), and IgG1 as well as IgG2a antibodies were similar to those elicited by the wild type antigen (fig. 5C). Consistent with these findings, ex-vivo recall assays demonstrated the presence of IFNγ-producing spleen cells after restimulation with both, recombinant protein or CTL peptide (fig. 5D). Together, these findings demonstrate that the enzyme activity of βGal does not account for the Th1 response of βGal gene gun vaccines.

### A gene gun vaccine encoding a βGal-OVA fusion protein induced IgG2a against OVA

**Figure 6:**
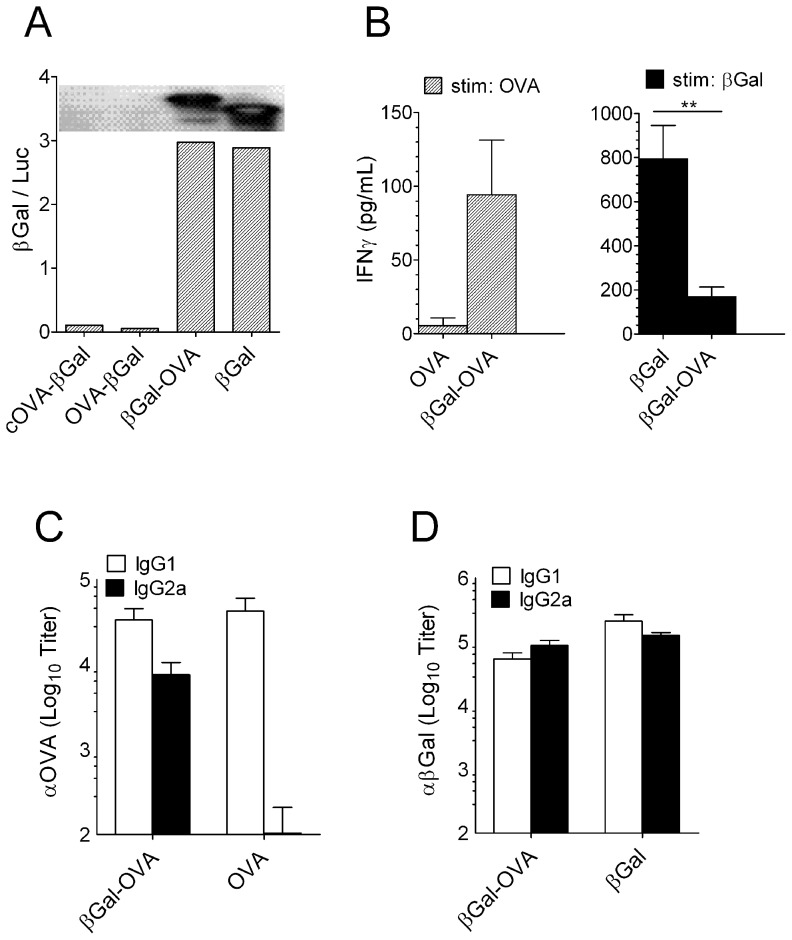
Gene gun immunization with a βGal-OVA fusion construct elicited IgG2a against OVA. (A) Enzymatic βGal activity in BHK21 cells transfected with, either, βGal or 3 different fusion constructs: “cytoplasmic” OVA with a deletion of AA 20–145 fused to the N-terminus of βGal (cOVA-βGal), full length OVA fused to, either, the N-terminus (OVA-βGal) or the C-terminus of βGal (βGal-OVA). Inset: Western blot of BHK21 cells transfected with the indicated plasmids and developed with anti-βGal antiserum. (B) IFNγ production by spleen cells from B6 mice (n = 5) gene gun-immunized 3× at 2 week intervals with the fusion construct pCI-βGal-OVA, measured by cytokine ELISA of culture supernatants after 48 hrs of restimulation in-vitro with OVA (left) or βGal (right). Mice immunized with pCI-OVA (left) or pCI-βGal (right) were included for comparison. IFNγ in non-stimulated medium controls were below detection limits (not shown). (C) βGal-specific and (D) OVA-specific IgG isotypes in sera of mice shown in (B).

We wondered whether proteins that induced only IgG1 in response to gene gun immunization would behave autonomously when fused to the IgG2a-inducing antigen βGal. To test this, we generated expression plasmids for various fusion constructs joining OVA to βGal. For full-length fusions, βGal-OVA or OVA-βGal, the full length coding sequence of OVA was fused, either, to the 3′ end or the 5′ end of full length βGal, respectively. cOVA-βGal was constructed by fusing an N-terminally truncated version of OVA that lacked the secretory signal peptide [37] to the 5′ end of βGal. By transiently transfecting BHK cells we found that only βGal-OVA was expressed with an efficacy and/or stability that was comparable to that of βGal. The other two constructs were not detectable by western blot analysis and βGal enzyme activity (fig. 6A). Gene gun immunization with pCI-βGal-OVA but not pCI-OVA induced OVA-specific Th1 cells as evidenced by IFNγ secretion after re-stimulation of spleen cells with OVA protein. Conversely, the βGal-specific Th1 response in mice immunized with the fusion construct was lower than in pCI-βGal immunized animals (fig. 6B). Consistent with this, immunization with the fusion construct induced IgG2a not only against βGal but also against the fusion partner, OVA (fig. 6C, D).

### βGal does not act as a Th1-polarizing modulator for other gene gun vaccines

**Figure 7:**
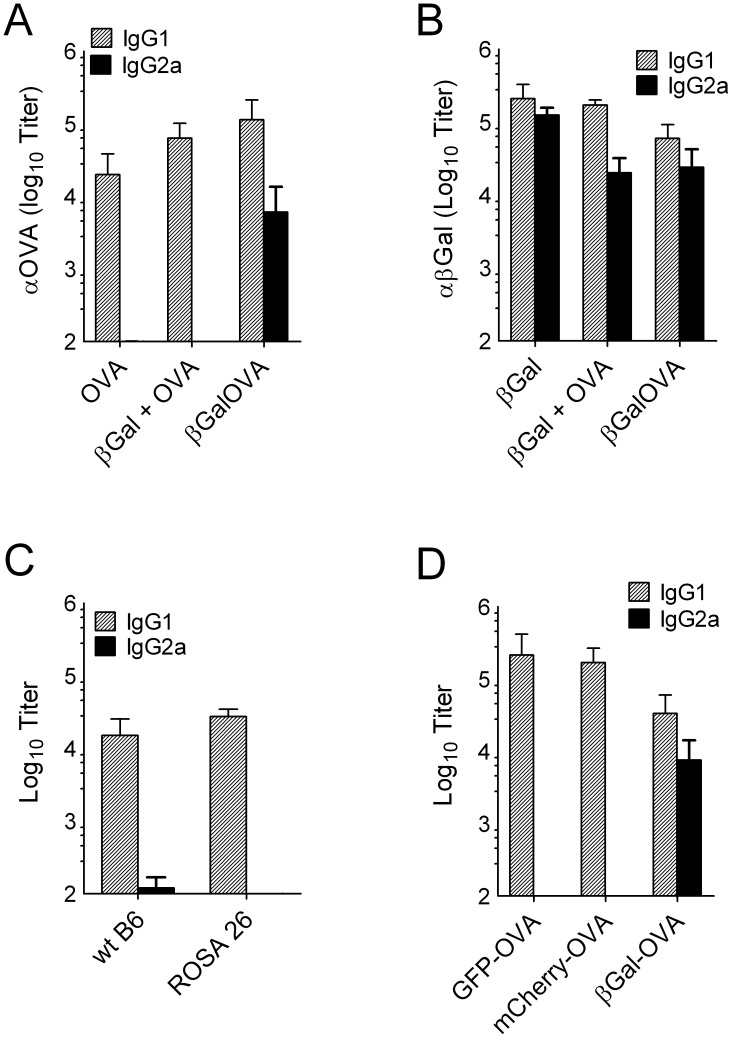
βGal is not a Th1 adjuvant for other antigens. (A) OVA-specific and (B) βGal-specific IgG in B6 mice (n = 5) gene gun-immunized with the fusion construct pCI-βGal-OVA or a mixture of pCI-OVA and pCI-βGal plasmids co-precipitated onto gold particles. For reference, groups of mice immunized with pCI-OVA (A) or pCI-βGal (B) were included. (C) OVA-specific IgG isotypes in B6 wild type mice or in mice constitutively expressing βGal (ROSA26), 2 weeks after 2 gene gun immunizations with pCI-OVA administered 2 weeks apart. (D) Serum IgG isotypes in B6 mice (n = 5) 2 weeks after two rounds of gene gun immunization, separated by a 2 week interval, with plasmid constructs in that secretion of OVA was prohibited by fusion to the C-terminus of, either, GFP, mCherry or βGal.

Because βGal promoted the induction of IgG2a against OVA when both antigens were fused to each other, we hypothesized that βGal might act as a Th1-polarizing immune modulator in general. However, when mice were gene gun-immunized with pCI-βGal and pCI-OVA, either at non-overlapping abdominal skin areas (not shown) or as a plasmid mixture co-immobilized on the same gold particles, both antigens induced their characteristic IgG isotype spectrum independently of each other (fig. 7A, B). Moreover, pCI-OVA did not induce IgG2a or IFNγ in ROSA26 mice, a transgenic mouse strain that expresses a βGal-neomycin-phosphotransferase fusion protein constitutively in all cells (fig. 7C). The reluctance of pCI-OVA gene gun vaccines to induce IgG2a was not simply due to the fact that OVA is naturally secreted from cells whereas βGal is retained in the cytosol. Fusion of OVA to the C-terminus of GFP or mCherry also prevented secretion from transfected cells. Despite this, gene gun vaccines encoding such fusion proteins were not able to induce IgG2a (fig. 7D).

## Discussion

In the present study, we provide evidence that E. coli βGal, when expressed by skin cells after gene gun immunization, induces an atypical antibody response and examine functional and structural requirements of the antigen to accomplish this. The influence of structural features of gene gun-encoded antigens on immunogenicity and T cell activation has been recognized before. E.g., fusion of weakly immunogenic HIV-gp120 fragments to the highly immunogenic hepatitis surface antigen elicited increased humoral and cellular immune reactions against HIV in mice and robust CTL in rhesus macaques [48], [49]. Such data have far-reaching implications for vaccine design, but the aim of these studies was not the investigation of underlying mechanisms.

In mice, gene gun vaccines usually induce Th2 reactions, as indicated by the predominant, sometimes virtually exclusive, appearance of the Th2-associated antibody isotype IgG1 [21], [22], [23]. However, this may not be the case in other species. E.g., rhesus macaques reacted to a gene gun vaccine encoding HIVgp160 with a balanced Th1/2 response [50] and, in human volunteers, a hepatitis B gene gun vaccine induced cellular responses dominated by IFNγ-secreting Th1 cells [51]. Even in mice, there are antigens that do not elicit a pure Th2 response after gene gun immunization. One such example is the circumsporozoite protein (CSP) from *plasmodium berghei* malaria parasites. Gene gun vaccines encoding CSP elicited, both, IgG1 as well as the Th1-dependent antibody isotype IgG2a. However, this was strongly influenced by the immunization regimen. In particular, IgG2a occurred only after repetitive vaccine administrations and increased with longer intervals between immunizations. However, even under optimal conditions, only a fraction of individuals in a group elicited IgG2a [52]. Compared to that, βGal as a gene gun vaccine is a particularly strong inducer of Th1 reactions. It induced similar serum titers of IgG2a/c and IgG1, not only in B6 but also BALB/c, which is a more Th2-biased strain as deduced from other models [53], [54], [55]. With βGal, the balanced isotype profile was elicited in all individuals immunized. IgG2a appeared early after the priming dose, the balanced isotype ratio remained stable over at least two months after the last immunization, and was obtained even with vaccines containing 10fold less plasmid.

As expected, the Th1 response induced by βGal gene gun vaccines proceeded along the classical pathway, because mice deficient in, either, IL12 or IFNγ were unable to produce IgG2a antibodies.

Therefore, βGal gene gun vaccines, but not such encoding other antigens, should be able to induce IL12 secretion by antigen-presenting DC. Unlike many microbial compounds that induce DC maturation and the production of the Th1 master cytokine IL12, host cell-expressed βGal is not a Th1 adjuvant in this traditional sense. Molecular activators of gene gun-induced immune reactions have not been clearly identified. It is possible that microbial compounds introduced by penetrating gold particles, plasmid DNA shot into skin cells, or host cell components released from damaged cells are involved in immune activation. However, it is not very likely that these factors are responsible for Th1 polarization of the βGal response because they are the same for Th2 polarizing antigens. Also, the Th1 response to βGal was unaltered in Tlr4-deficient mice.

Thus, because both, the material delivered as well as the vaccination procedure are the same for all antigens, we hypothesized that any deviation in the resulting immune reaction should be determined by the antigen expressed in the host's cells. In the case of βGal, one possibility is that cleavage of galactosyl residues from immunologically relevant molecules could be involved in the immune modulating activity. Galectins, a family of lectins with specificity for β-galactosides have been implicated in many biological functions including innate and adaptive immunity [47], [56]. However, this was apparently not a key factor for the Th1 response to βGal because a gene gun vaccine with a point mutation in the catalytic center was still able to induce IgG2a.

A second possibility how βGal could modulate T cell polarization is triggering of a known or unknown receptor on DC. Antigens with immune modulating activity have been identified before, such as the house dust mite allergen Der p 2 that has structural homology to the LPS-binding co-receptor of Tlr-4, MD-2 [13]. The monomeric polypeptide of βGal comprises five well-defined structural domains, and a receptor-triggering motif could reside in a one of them. However, gene gun vaccines encoding individual domains of βGal were unable to induce Th1 reactions. Moreover, even the almost complete polypeptide, with just a short deletion at the N-terminus, was little immunogenic and elicited a pure Th2 response. *In-silico* structure prediction analysis was consistent with the assumption that structures were retained in isolated domains as well as in the N-terminally truncated polypeptide. For the latter, circular dichroism spectroscopy revealed that secondary structure composition was identical to that in the native tetrameric protein. Also, isolated domains as well as the deletion mutant were recognized by antibodies raised against the native protein. Nevertheless, we cannot strictly exclude the possibility that critical motifs could have been distorted sufficiently in these fragments to prevent the hypothesized interaction with DC receptors. Unlike the native βGal tetramer, the deletion mutant showed increased tendency to form high molecular weight aggregates that were also more resistant DC-derived microsomal proteases. It is conceivable that this could reduce availability of antigenic peptides to be presented on MHC molecules. Reduced density of MHC:peptide complexes on DC might in turn account for the decreased immunogenicity and, by lowering MHC:TCR avidity [57], [58], also for the observed Th2 response with this mutant.

Alternative approaches to investigate the potential nature of βGal as a molecular adjuvant were, therefore, focused on the native tetrameric protein. If βGal is a molecular adjuvant it should be able to confer Th1 reactivity to other antigens. However, transgenic mice that express βGal constitutively in all cells also failed to induce IgG2a when immunized with an OVA gene gun vaccine. Likewise, co-immunization of βGal with OVA encoded on separate plasmids, but with both plasmids immobilized on the same particles, failed to skew the anti-OVA reaction towards Th1. Only when the OVA coding sequence was directly fused to the βGal reading frame, mice elicited a Th1 response against OVA epitopes. In turn, IFNγ production by βGal-specific Th cells was reduced. This was not simply due to the cytosolic retention of the otherwise secreted OVA, as cytosolic retention by fusion to EGFP did not lead to Th1 reactions against OVA. Thus, the Th1-promoting activity of βGal and βGal fusion proteins applies only to these molecules themselves but does not affect separate antigens, even when produced in the same cells.

Taken together, all above data dismiss the hypothesis of βGal as a molecular adjuvant in the classical sense. What then could be the origin of the Th1 response to βGal gene gun vaccines? Noteworthy, a Th1 response was also elicited with gene gun vaccines that restricted βGal expression to keratinocytes, suggesting that direct transfection of APC is not required (unpublished data). Gene gun bombardment predominately transfects epidermal cells, the majority of which is keratinocytes (KC) that are known to entertain intensive communication with the immune system [59], [60], [61]. In view of this it is tempting to speculate whether KC might differentially pass antigens on to different DC subsets that, in turn might be specialized to polarize Th cells in different ways [62], [63], [64]. Indeed, we observed markedly different immune reactions with βGal gene gun vaccines in the presence or absence of langerin^+^ cells [40]. Also, we have now evidence that the immunogenicity of different antigens, either rises or falls with the presence or absence of distinct DC subsets (in preparation).

In conclusion, our data dismiss the hypothesis of βGal as an immune modulating activity. However, the structural integrity of the molecule, but not its enzymatic activity, is an essential prerequisite for the induction of Th1 immunity. Protective immunity does not only rely on sufficient strength but also on the appropriate type of an immune response. The data presented here demonstrate that even minor modifications in an antigen's amino acid sequence can cause fundamental quantitative as well as qualitative changes in the immune response. Bearing such effects in mind might therefore also aid in vaccine design. Clarification of the underlying cell biological mechanisms, particularly those of the initial steps of antigen transfer to DC, might provide new insights into the initial steps of skin-borne antigens in the induction of immune reactions and contribute to our understanding of skin immunity.
